# Diagnostic Utility of Serum Leucine-Rich α-2-Glycoprotein 1 for Acute Appendicitis in Children

**DOI:** 10.3390/jcm12072455

**Published:** 2023-03-23

**Authors:** Goran Tintor, Miro Jukić, Daniela Šupe-Domić, Ana Jerončić, Zenon Pogorelić

**Affiliations:** 1 Department of Plastic Reconstructive and Aesthetic Surgery, University Hospital of Split, 21000 Split, Croatia; gogitintor@gmail.com; 2 Department of Surgery, School of Medicine, University of Split, 21000 Split, Croatia; mirojukic.mefst@gmail.com; 3 Department of Pediatric Surgery, University Hospital of Split, 21000 Split, Croatia; 4 Department of Medical Laboratory Diagnostics, University Hospital of Split, 21000 Split, Croatia; daniela.supedomic@gmail.com; 5 Department of Health Studies, University of Split, 21000 Split, Croatia; 6 Department of Research in Biomedicine and Health, School of Medicine, University of Split, 21000 Split, Croatia; ajeronci@mefst.hr

**Keywords:** acute appendicitis, children, serum, biomarker, leucine-rich α-2-glycoprotein, LRG1

## Abstract

Purpose: The aim of this study is to assess the diagnostic utility of serum leucine-rich α-2-glycoprotein 1 (LRG1) in pediatric patients with acute abdominal pain, admitted to the emergency surgical unit, in order to make a prompt and accurate diagnosis of acute appendicitis. Patients and methods: Pediatric patients older than 5 years of age who presented to the emergency department from 15 October 2021 to 30 June 2022 with acute abdominal pain and suspected acute appendicitis were prospectively recruited in the study. Demographic and clinical data, as well as operative and postoperative data, were recorded. A total of 92 patients were equally distributed into two groups: children with acute appendicitis who underwent laparoscopic appendectomy and non-appendicitis patients, presenting with non-specific abdominal pain. LRG1 levels were determined using a commercially available LRG1 enzyme-linked immunosorbent assay (ELISA) kit. Serum LRG1 levels, as well as other inflammatory markers (white blood cell count (WBC), C-reactive protein (CRP) and absolute neutrophil count) were compared between groups. Results: The median level of LRG1 in serum was significantly higher in the group of children with pathohistologically confirmed acute appendicitis than in the control group, at 350.3 µg/mL (interquartile range (IQR) 165.2–560.3) and 25.7 µg/mL (IQR 14.7–36.8) (*p* < 0.001), respectively. Receiver operating characteristic area under the curve for LRG1 from serum was 1.0 (95% CI 0.96–1.00; *p* < 0.001) and the value of >69.1 µg/mL was found to perfectly separate acute appendicitis cases from controls. Additionally, as expected, each of the examined laboratory inflammatory markers provided a significantly higher values in the acute appendicitis group compared to the control group: WBC 14.6 × 10^9^/L (IQR 12.7, 18.7) vs. 7.0 × 10^9^/L (IQR 5.4, 9.0) (*p* < 0.001), CRP 16.3 mg/dL (IQR 6.9, 50.4) vs. 2.2 mg/dL (IQR 2, 2) (*p* < 0.001) and absolute neutrophil count 84.6% (IQR 79.5, 89.0) vs. 59.5% (IQR 51.5, 68.6) (*p* < 0.001). Conclusions: LRG1 in the serum was found to be a promising novel biomarker, with excellent differentiation of acute appendicitis from non-appendicitis cases in children presenting with non-specific abdominal pain.

## 1. Introduction

Despite being the most common surgical emergency in the pediatric population, a correct diagnosis of acute appendicitis or its exclusion remains difficult and frequently expensive [1,2,3]. In support of that, there are enough data to demonstrate substantial overlap in the clinical characteristics of acute appendicitis and other prevalent pediatric diseases, such as acute gastroenteritis, mesenteric lymphadenitis, torsion of intraabdominal organs, Meckel’s diverticulitis, Crohn’s disease, enterobiasis, etc. [4,5,6]. Up to 30% of children who are brought to a pediatric surgical emergency department with acute abdominal pain are found to have acute appendicitis [7,8]. Children, especially those under the age of five, are reported to typically have considerably higher perforation rates than adults [9,10,11,12,13]. Although appendectomy is the gold standard of treatment for acute appendicitis, recent studies indicate that uncomplicated acute appendicitis may also be treated conservatively [14,15,16]. A rushed diagnosis can precipitate “negative” appendectomy [17]. On the contrary, failure to make an accurate diagnosis may result in perforation [18]. Consequently, with prompt diagnosis and suitable treatment for acute appendicitis, various complications, primarily appendiceal perforation, may be prevented [19,20]. Conventionally, patients’ medical history, physical examination and laboratory hematological biomarkers have been used to diagnose acute appendicitis, but with variable success [21,22]. Standard laboratory measurements incorporate complete blood count, white blood cell count (WBC) and C-reactive protein (CRP) [23]. When assessing a diagnosis of acute appendicitis in a pediatric population displaying acute abdominal pain, CRP complements the history and physical examination [24]. If the CRP level is below 10 mg/L and symptoms have been evident for longer than 48 h, acute appendicitis is least likely to be the cause [22,25]. To enhance diagnostic performance, clinical prediction scores have been introduced. The Alvarado score and Pediatric appendicitis score (PAS) have been frequently applied, but they appeared to be less beneficial in the youngest children [26,27,28,29,30]. In correlation with the Alvarado score and PAS, the appendicitis inflammatory response (AIR) score demonstrated higher sensitivity and specificity [31]. Furthermore, radiological techniques, such as ultrasound, computed tomography (CT) and even magnetic resonance imaging, have become heavily relied upon [32,33,34]. However, extensive use of diagnostic imaging has been severely restricted by a variety of factors, including immediate accessibility, cost, exposure of children to radiation from CT scans and the national guidelines [35,36]. Notable studies are focused on utilizing new biomarkers of inflammation that are accessible, easily detected and affordable in order to accurately diagnose acute appendicitis [1,37,38,39,40,41,42,43,44,45].

To date, several biomarkers, such as fibrinogen, red cell distribution width, mean platelet volume, interleukins, CRP, procalcitonin, 5-hydroxyindoleacetic acid, hyponatremia or hyperbilirubinemia, have been investigated for acute appendicitis with varying diagnostic accuracy in published studies [1,21,22,46]. A novel protein biomarker known as leucine-rich alpha-2-glycoprotein 1 (LRG1) has recently been found in the diseased appendices and has been elevated in the serum, urine and saliva of patients with acute appendicitis [42,43,44,45,46,47]. Even though its exact physiological function is relatively unclear, recent studies have confirmed that LRG1 is involved in cell adhesion and signal transduction [47,48]. It has been demonstrated that LRG1 serves as an acute-phase protein deposited by differentiating neutrophils, hepatocytes and venules of the mesentery [49].

The objective of our study is to assess the diagnostic utility of LRG1 in children who presented to the emergency department with acute abdominal pain.

## 2. Materials and Methods

### 2.1. Study Design and Setting

This prospective, controlled study was performed at the Department of Pediatric Surgery of University Hospital of Split, Croatia. Children aged 5 to 17 who presented to the emergency department with acute abdominal pain and were being evaluated for suspected appendicitis were eligible for registration from 15 October 2021 to 30 June 2022. Patients who were known to be pregnant, had an invasive abdominal procedure or had a long-term illness or cancer were excluded from our study. 

The study was approved by the local institutional Ethics Committee of our hospital (Reference: 500-03/21-01/40; Date of approval: 1 April 2021). Before taking part in the research, each patient’s parent or other legally permitted relative gave their written consent. The study was registered in the ClinicalTrials.gov registry under identifier NCT05093660.

### 2.2. Study Protocol

Complete medical history, patient demographics (age, sex, weight and height), clinical signs and symptoms (duration of symptoms, right lower quadrant abdominal pain, rebound tenderness and body temperature), information about surgery, clinical type of appendicitis (catarrhal, phlegmonous, gangrenous and perforated), length of surgical procedure with its potential complications and postoperative data (length of hospitalization and pathohistological analysis) were all recorded. A routine blood test was performed to quantify the CRP level, neutrophil percentage (Neu%) and WBC. The appendicitis inflammatory response (AIR) score was implemented to evaluate seven distinct prognostic variables. For all patients involved in this study, abdominal and pelvic ultrasounds were necessary. The patients were split into two groups. Patients with acute appendicitis who underwent laparoscopic appendectomy were included in the acute appendicitis group (*n* = 46). All patients in the first group underwent a three-access laparoscopic appendectomy, as detailed in our previous research [50]. Patients who presented with non-specific abdominal pain and in whom acute appendicitis could be ruled out by diagnostic procedures made up the non-appendicitis group (*n* = 46). These patients were admitted to the hospital for observation.

### 2.3. Blood Collection and Analysis for Standard Inflammatory Serum Biomarkers

Blood was drawn from the patient’s brachial veins and placed in a vial containing clot activator and a vial containing the anticoagulant tripotassium ethylenediaminetetraacetic acid (K3 EDTA). Immediately after blood collection, the vials were taken to the department of medical laboratory diagnostics. Serum from the vial with clot activator was used to measure CRP levels. Blood collected in a vial containing K3 EDTA was used to analyze white blood cell count and total neutrophils. An analysis of leukocyte and total neutrophil counts was performed in a routine laboratory using a hematological blood analyzer (Siemens Advia 2120 Hematology Analyzer, Bayer, Germany). A CRP assay was performed by the immunoturbidimetric method using the Cobas C702 chemistry analyzer (Roche, Rotkreuz, Switzerland).

### 2.4. Serum LRG1 Collection and Analysis

Per analysis, 300 µL was the lower limit of blood volume considered necessary for each patient when determining the concentration level of LRG1 in serum. The samples were centrifuged at 4000× *g* for 10 min before being deposited at −80 °C. Prior to commencing the final analysis, the prepared samples were stabilized and homogenized at room temperature. A commercially available enzyme-linked immunosorbent assay (ELISA) kit (Immuno-Biological Laboratories Co., Ltd. International, Takara, Japan) was employed to undertake a quantitative analysis of Human LRG1 in serum according to the manufacturer’s instructions. In a solid phase sandwich ELISA, this kit contains two different kinds of highly specific antibodies. Tetramethylbenzidine dihydrochloride hydrate (Merck KGaA, Darmstadt, Germany) is used as a coloring agent. The level of LRG1 and the intensity of the color are directly correlated. With the provided dilution buffer, serum samples were diluted 1:1000. For additional calculations, acquired values were expressed in µg/mL. 

### 2.5. Final Diagnosis of Patients

The diagnosis of acute appendicitis was based primarily on the intraoperative findings. Approximately two to three weeks after surgery, the results of the histopathology analysis were completed, confirming the initial diagnosis. Only the cases with confirmed diagnosis by histopathology were included in acute appendicitis group. In cases where the appendicitis was not confirmed by histopathology, the samples were excluded from analysis. After at least 24 h of observation in a hospital setting, where no surgical procedure was necessary, it was ascertained that acute appendicitis was excluded. The researchers who analyzed the LRG1 level in serum were not given access to the definitive, histologically confirmed diagnosis.

### 2.6. Sample Size Calculation

A minimum of 82 subjects (41 per group), drawn from a consecutive sample of children who were admitted to a pediatric emergency department with a possible case of appendicitis, are needed to conduct the study, assuming a power of 80%, a significance level of 0.05 and an area under the curve (AUC) for the null hypothesis of 0.5 and 0.69 for the alternative hypothesis. To guarantee adequate power to account for potential dropouts culminating from negative appendectomies or conclusions about the variables used in the study, we aimed for a minimum of 46 subjects per group.

### 2.7. Statistical Analysis

The software SPSS 24.0 (IBM Corp, Armonk, NY, USA) was used for the statistical analysis. While the distributions of the quantitative data were described with mean and standard deviation or median and interquartile range, depending on the normality of the data, the distributions of the qualitative data were described with absolute and relative frequencies. The independent *t*-test or its non-parametric equivalent, the Mann–Whitney U test, were used to infer differences between patient groups after the D’Agostino–Pearson test was used to assess the normality of the data. To measure the usefulness of a diagnostic test based on LRG data, we performed a receiver operating characteristic (ROC) analysis using the methodology of deLong et al. [51], and used the area under the curve (AUC) and Youden index J to assess performance of the test. All *p*-values < 0.05 were considered as statistically significant. The optimal criterion for separation of groups that considers prevalence of disease is also calculated under assumption that overall, 7% of children presenting with abdominal pain have acute appendicitis [6].

## 3. Results

### 3.1. Baseline Characteristics and Clinical Data of the Patients

The demographic data of the two groups of patients are summarized in Table 1. There were no statistically significant differences between the two investigated groups of the patients in regard to age (*p* = 0.602), gender (*p* = 0.130), body weight (*p* = 0.861) and height (*p* = 0.876).

Intraoperative finding was positive for acute appendicitis in all of the cases from the acute appendicitis group (*n* = 46, 100%). The pathohistological analysis of removed specimens in the acute appendicitis group showed that the majority of the patients (*n* = 20; 43.5%) had phlegmonous appendicitis, while in 16 (34.8%) patients, gangrenous appendicitis was reported. Perforated gangrenous appendicitis was found in 10 (21.7%) patients.

### 3.2. LRG1 from Serum as an Biomarker of Acute Appendicitis

Serum LRG1 has been shown to be an excellent biomarker for acute appendicitis in children. In the group of children who had pathohistologically confirmed acute appendicitis, the median serum level of LRG1 was 350.3 µg/mL (IQR 165.2, 560.3), while the median LRG1 in the control group of patients was significantly lower and was 25.7 µg/mL (IQR 14.7, 36.8) (*p* < 0.001).

**Figure 1:**
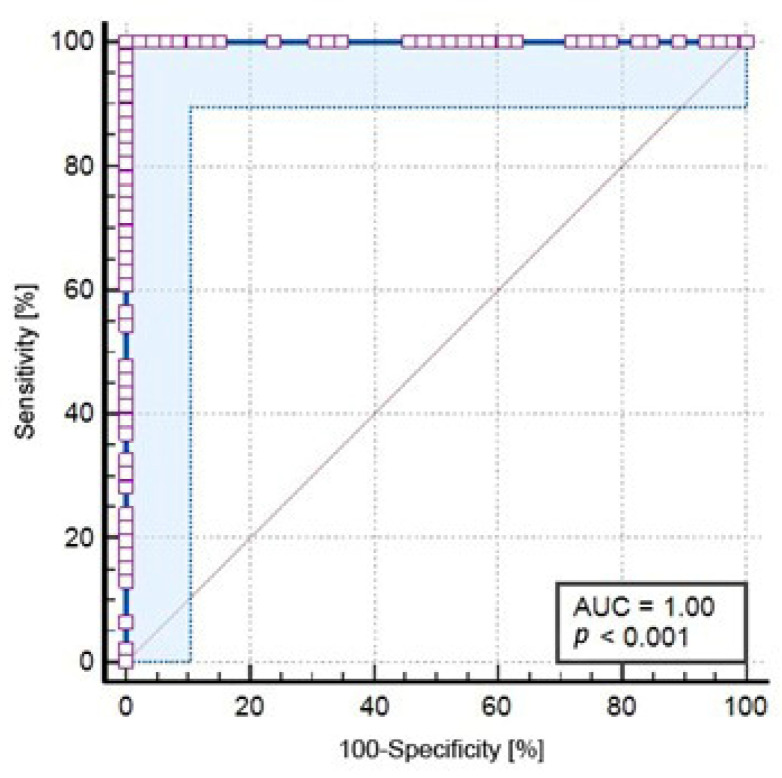
Receiver operating characteristic curve for LRG1 from human serum as a predictor of acute appendicitis (AUC = 1.00; 95% CI 0.96–1.00; *p* < 0.001).

Serum LRG1 values were completely separated between groups, i.e., there was no overlap between LRG1 values measured in the acute appendicitis group and controls. Consequently, both AUC and Youlden’s index J values for this variable were maximal: 1.0 (95% CI 0.96–1.0) i.e., 1.0 (95% CI > 0.99–1.0) (Figure 1). 

**Figure 2:**
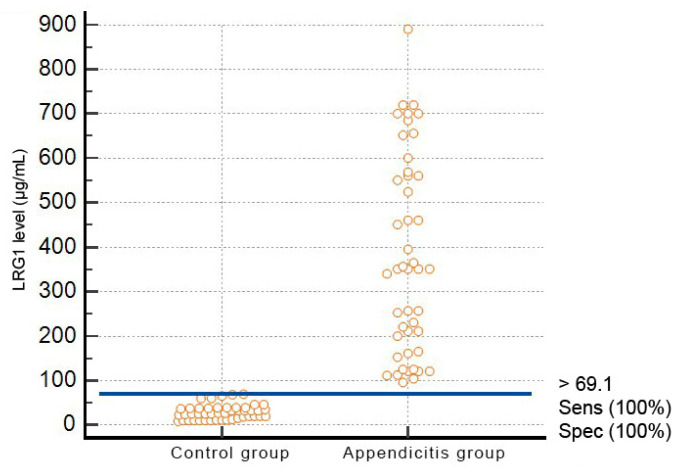
One hundred percent accurate separation of acute appendicitis vs. non-appendicitic cases based on the >69.1 µg/mL cut-off for LRG1 levels.

With a 7% prevalence of appendicitis in children assumed, a value of >69.1 µg/mL was determined as an optimal cut-off (Figure 2).

### 3.3. Other Factors Associated with Acute Appendicitis

In Table 2, clinical and laboratory data of the patients have been summarized. As expected, a significant increase of all investigated laboratory inflammatory markers in patients with acute appendicitis compared to those from control group was observed.

## 4. Discussion

Although diagnostic imaging provides high sensitivity and specificity, the predictive ability of diagnosing acute appendicitis is still heavily supported by clinical assessment. Prevailing inflammatory markers such as WBC and CRP are insufficient for establishing the diagnosis of appendicitis with a high level of specificity or sensitivity [24]. Due to the latter considerations, in order to confirm an initial, precise diagnosis of acute appendicitis and to assess its severity, recent scientific investigations aim to identify widely available and cost-effectively produced biomarkers. We identified the LRG1 level in serum as a promising diagnostic biomarker. The results of our study showed that LRG1 in the serum enabled a 100% accurate separation between acute appendicitis and controls at the determined threshold concentration of 69.1 µg/mL.

Due to the limited accuracy caused by immunoassay interference, Kentsis et al. recommended selected ion monitoring mass spectrometry over the commercially available serum LRG1 ELISA test, reaching AUC levels of 0.98–0.99 and 0.80, respectively [39]. Our results reinforce several recent publications that indicated a connection between an increased LRG1 level and the established diagnosis of acute appendicitis in the pediatric population. Kharbanda et al. prospectively evaluated 148 children with suspected acute appendicitis and found a significant elevation of serum LRG1 in patients with confirmed diagnosis of acute appendicitis compared to controls. Even though 100% sensitivity and negative predictive value were accomplished, LRG1 measured by ELISA reached an AUC of only 0.69 and showed a specificity of just 35% [37]. The results of the study implemented by Kakar et al. suggested not only a positive correlation of serum LRG1 with the severity of appendicitis but also an inversely proportional correspondence with patient recovery. At the selected threshold concentration of 51.69 µg/mL, serum LRG1 exhibited 91.1% specificity and 93.8% sensitivity, with an AUC of 0.95. Serum LRG1 levels presented in the study might have been compromised by the therapy that was administered before sample collection. Furthermore, due to the restrictions on the number of participants admitted to this study, the control group consisted of the patients with different surgical conditions, such as fractures, testicular torsion, etc. and the patients with non-specific abdominal pain were not included [43]. On the contrary, to differentiate acute appendicitis from non-specific abdominal pain, in our control group, we included children who had non-specific abdominal pain but in whom the diagnosis of acute appendicitis had been ruled out by diagnostic procedures. On the other hand, the literature review contains inconsistencies, with Demirci et al. and Lontra et al. reporting an insignificant discrepancy between adults with and without acute appendicitis in aspects of the serum LRG1 level [38,44]. The latter study’s lack of a clinically significant finding is attributed to the relatively small sample size of only 28 patients in total [44].

Salo et al. showed improved outcomes when they combined the pediatric appendicitis score with LRG1 in urine [41]. This was in line with the study conducted by Yap et al., where only a slight improvement in the diagnostic utility of urine LRG1 compared to other conventional inflammatory parameters was observed [1]. This could be clarified by LRG being secreted in reaction to any inflammatory stimulation. As a matter of fact, a wide range of diseases, including retinoblastoma, respiratory inflammatory disorder, hepatocellular carcinoma, ulcerative colitis and heart failure, have been linked to elevated serum LRG1 levels in numerous clinical reports [52,53]. O’Donnell et al. discovered that LRG1 played a role in early neutrophilic granulocyte differentiation. By attaching to cytochrome C, LRG1 promotes preservation and protection of lymphocytes in the tissue of the appendix [54]. Additionally, Wang et al. showed that LRG1 is involved in aberrant neovascularization, which could exacerbate inflammatory reactions and further increase neutrophil secretion of LRG1 [55].

When we compare these results with our previous study on saliva-measured LRG1, in the same cohort of patients, we can clearly see that serum-measured LRG1 has a significantly higher sensitivity for detecting acute appendicitis in the pediatric population than saliva-measured LGR1. We found that salivary LGR1 has a good AUC parameter and significantly higher levels in patients with acute appendicitis compared with controls, but its usefulness in a patient population presenting to a hospital emergency department with abdominal pain is questionable because the sensitivity for detecting acute appendicitis is only 36% [56].

The current study had several limitations. It was designed as a single-center study. Furthermore, only one type of commercially available ELISA kit was employed. Since appendicitis tends to have a different pathophysiology in children under the age of five [13], those patients were not included in the study. Accordingly, this age group should be the subject of additional research.

## 5. Conclusions

When combined with a clinical suspicion of acute appendicitis, the results suggest that LRG1 levels in serum have a high biomarker potential for confirming the acute appendicitis diagnosis in pediatric patients. Well-designed prospective diagnostic comparative effectiveness studies are essential to stimulate research focus on the diagnostic value of serum biomarkers for a wide range of causes of acute abdominal pain other than acute appendicitis.

## Figures and Tables

**Table 1:** Demographic data of the patients.

Variables	Group				*p*
	Acute Appendicitis (*n* = 46)		Non-Appendicitis (*n* = 46)		
	Mean	SD	Mean	SD	
Age (years)	11.4	3.3	11.8	3.0	0.602 *
Body weight (kg)	47.4	17.1	48.0	16.1	0.861 *
Body height (cm)	154.3	19.6	154.9	16.3	0.876 *
Gender	*n*	%	*n*	%	
Male	33	71.7	13	28.3	0.130 ^‡^
Female	26	56.5	20	43.5	

SD—Standard Deviation; * Independent samples *t*-test; ^‡^ Chi-square test.

**Table 2:** Clinical and laboratory data of the patients.

Variables	Group				p *
	Acute Appendicitis (*n* = 46)		Non-Appendicitis (*n* = 46)		
	Median	IQR	Median	IQR	
Duration of symptoms (h)	25	(18, 36)	32.5	(24, 50)	0.031
AIR score	9	(7, 10)	3	(3, 4)	<0.001
Body temperature (°C)	37.3	(36.9, 37.6)	36.8	(36.6, 36.9)	<0.001
WBC (×10^9^/L)	14.6	(12.7, 18.7)	7.0	(5.4, 9.0)	<0.001
CRP (mg/dL)	16.3	(6.9, 50.4)	2.2	(2, 2)	<0.001
Neutrophil count (%)	84.6	(79.5, 89.0)	59.5	(51.5, 68.6)	<0.001
LRG1 in serum (µg/mL)	350.3	(165.2, 560.3)	25.7	(14.7, 36.8)	<0.001
Duration of surgery (min)	21	(18, 30)	-	-	-
LOS (days)	2	(1, 3)	2	(2, 2)	0.856

IQR—interquartile range; AIR—appendicitis inflammatory response; WBC—white blood cells count; CRP—C-reactive protein; LRG1—leucine-rich α-2 glycoprotein 1; LOS—length of hospital stay; * Mann–Whitney U test.

## Data Availability

The data presented in this study are available upon request of the respective author. Due to the protection of personal data, the data are not publicly available.
